# Blood‐based ATN biomarkers among Hispanics/Latinos across depressive symptom subscales: Findings from the Study of Latinos‐Investigation of Neurocognitive Aging

**DOI:** 10.1002/alz70856_104721

**Published:** 2026-01-07

**Authors:** Natasha Z. Anita, Wassim Tarraf, Ariana M Stickel, Kevin A Gonzalez, Freddie Márquez, Linda C Gallo, Melissa Lamar, Carmen R Isasi, Paola Filigrana, Sonya Kaur, Christian Agudelo, Krista M Perreira, Martha L Daviglus, Fernando Daniel Testai, Douglas R. Galasko, Hector M González

**Affiliations:** ^1^ University of California, San Diego, La Jolla, CA, USA; ^2^ Wayne State University, Detroit, MI, USA; ^3^ San Diego State University, San Diego, CA, USA; ^4^ Rush Alzheimer's Disease Center, Rush University Medical Center, Chicago, IL, USA; ^5^ Albert Einstein College of Medicine, Bronx, NY, USA; ^6^ University of Miami Miller School of Medicine, Miami, FL, USA; ^7^ Evelyn F. McKnight Brain Institute, Miami, FL, USA; ^8^ University of North Carolina, Chapel Hill, NC, USA; ^9^ University of Illinois at Chicago, Chicago, IL, USA; ^10^ UIC, Chicago, IL, USA; ^11^ Shiley‐Marcos Alzheimer's Disease Research Center, University of California, San Diego, CA, USA

## Abstract

**Background:**

Depressive symptoms are a modifiable risk factor for dementia, yet underlying mechanisms remain unclear. Depression is a heterogeneous condition, encompassing a wide range of symptoms, and it may be valuable to identify specific symptom patterns that are most associated with an increased risk of cognitive impairment and blood‐based biomarkers. Here, we investigated the associations between Visit 1 depressive symptoms and plasma Amyloid‐Tau‐Neurodegeneration‐Inflammation or ATN(I) biomarkers 7‐years later in a diverse cohort of middle‐aged and older Hispanic/Latino individuals, a population often underrepresented in dementia research.

**Method:**

Participants recruited into the Hispanic Community Health Study/Study of Latinos (HCHS/SOL; Visit 1 conducted between 2008–2011) and its ancillary study, Study of Latinos‐Investigation of Neurocognitive Aging (SOL‐INCA; Visit 2 conducted between 2015‐2018) were screened for somatic, negative affective, and anhedonia symptoms of depression at Visit 1 (Center for Epidemiological Studies‐Depression Scale, CESD‐10). Plasma ATN(I) biomarkers included beta‐amyloid 42/40 (Aβ42/40 ratio, *n* = 5,536), phosphorylated tau181 (*p*‐tau181, *n* = 5,713), neurofilament light chain (NfL, *n* = 5,866), and glial fibrillary acidic protein (GFAP, *n* = 5,686) measured at Visit 2 7‐years later (Quanterix Simoa HD‐X Analyzer). Survey‐based generalized linear regressions measured associations between continuous depressive symptoms (somatic, negative affective, and anhedonia subscales, respectively) and plasma ATN(I) biomarkers 7‐years later, adjusting for age, sex, Hispanic/Latino heritage, body mass index, APOE status, education, diabetes, cardiovascular disease risk factors (smoking, dyslipidemia, hypertension), antidepressant use, and field center.

**Result:**

Higher somatic symptoms at Visit 1 were associated with higher ptau‐181 (β= 0.03, 95% CI [‐0.05; 0.12]), higher NfL (β= 1.30, CI [‐0.52;3.12]), and lower GFAP (β= ‐2.95, CI [‐8.36;2.46]) 7‐years later, albeit not significantly. Higher anhedonia and negative affective symptoms were associated with lower ptau‐181 (β= ‐0.05, CI [‐0.11;0.01] and β= ‐0.05, CI [‐0.11;0.02], respectively), higher NfL (β= 0.02, CI [‐0.79;0.83] and β=0.45, CI [‐0.78; 1.69] respectively), and lower GFAP (β= ‐2.71, CI [‐6.30;0.88] and β= – 2.37, CI [‐6.49;1.74]).

**Conclusion:**

The current work suggests that different depressive symptoms may uniquely relate to distinct ATN(I) biomarkers. Future studies will examine the potential impact of chronic depressive symptoms as this longitudinal cohort ages as well as neurocognitive outcomes, providing a deeper understanding of the pathways linking depression to cognitive decline.

